# Supplementary material for: Dietary breadth is positively correlated with venom complexity in cone snails
Source: BMC Genomics. 2016 May 26;17:401. doi: 10.1186/s12864-016-2755-6 (PMC4880860; doi:10.1186/s12864-016-2755-6)
Supplement: Additional file 1: Table S1. — Sequencing and assembly statistics for each species. Values were calculated using all transcripts assembled from all iterations of Trinity. (PDF 59 kb) [file 12864_2016_2755_MOESM1_ESM.pdf]

Table S1. Sequencing and assembly statistics for each species. Values were calculated using all transcripts assembled from all iterations of Trinity.

Species	# of reads	# of contigs	n50	Average contig length	Total length assembled (bases)
arenatus	27,258,786	39198	576	497.37	19495780
californicus	26,639,152	88052	701	563.53	49619898
coronatus	24,688,386	33924	538	482.27	16360369
ebraeus	22,790,706	49854	612	520.89	25968607
imperialis	24,788,128	28878	613	517.78	14952318
lividus	26,203,686	51759	589	509.29	26360522
marmoreus	26,116,312	47371	635	532.81	25239624
quercinus	22,956,900	49429	624	527.69	26083140
rattus	28,843,008	46244	616	522.14	24146003
sponsalis	28,987,708	41604	573	501.67	20871502
varius	28,480,236	56390	643	533.4	30078380
virgo	21,635,650	35958	591	512.26	18419985
