# Supplementary material for: Dietary breadth is positively correlated with venom complexity in cone snails
Source: BMC Genomics. 2016 May 26;17:401. doi: 10.1186/s12864-016-2755-6 (PMC4880860; doi:10.1186/s12864-016-2755-6)
Supplement: Additional file 2: Table S2. — Conotoxin diversity by gene family for each species sequenced in this study. Each value represents the number of unique predicted mature toxins. Values in parentheses represent the number of unique precursor peptides. (PDF 74 kb) [file 12864_2016_2755_MOESM2_ESM.pdf]

Table S2. Conotoxin diversity by gene family for each species sequenced in this study. Each value represents the number of unique predicted mature toxins. Values in parentheses represent the number of unique precursor peptides.

Superfamily	*arenatus*	*californicus*	*coronatus*	*ebraeus*	*imperialis*	*lividus*	*marmoreus*	*quercinus*	*rattus*	*sponsalis*	*varius*	*virgo*
A	11 (14)	-	7 (8)	-	3 (3)	18 (21)	1 (1)	7 (8)	-	3 (3)	12 (16)	3 (5)
B1	10 (13)	-	8 (9)	1 (2)	-	7 (9)	-	-	-	2 (2)	2 (2)	1 (1)
B2	1 (2)	-	-	-	1 (1)	2 (2)	1 (1)	1 (2)	-	-	2 (2)	-
B4[r]	-	-	1 (1)	-	-	6 (11)	-	2 (6)	2 (2)	1 (1)	7 (8)	-
C	1 (1)	-	-	1 (1)	-	3 (3)	-	-	1 (1)	-	-	-
con-ikot-ikot	21 (26)	-	19 (21)	1 (1)	-	13 (14)	-	3 (4)	16 (17)	9 (9)	6 (8)	1 (1)
conkunitzin	11 (13)	5 (5)	9 (10)	2 (2)	-	10 (11)	1 (1)	-	6 (6)	12 (12)	2 (2)	1 (1)
conodipine	4 (4)	2 (3)	3 (3)	5 (5)	-	-	-	2 (2)	2 (2)	7 (7)	4 (4)	3 (3)
conohyal	-	1 (1)	-	-	-	-	-	-	-	-	-	-
conophysin	2 (2)	-	2 (2)	2 (2)	1 (1)	-	-	1 (1)	1 (1)	3 (3)	4 (4)	3 (4)
conoporin	-	9 (9)	8 (8)	1 (1)	2 (2)	2 (2)	-	-	8 (8)	-	7 (7)	-
D	1 (1)	-	1 (2)	1 (2)	1 (1)	-	-	-	4 (6)	3 (5)	-	-
Divergent_MKFPLLFISL	-	6 (6)	3 (4)	3 (4)	2 (2)	-	-	-	1 (1)	4 (5)	1 (1)	-
Divergent_MKLCVVIVLL	-	5 (6)	-	-	-	-	-	-	-	-	-	-
Divergent_MKLILTLLG	-	4 (4)	-	-	-	-	-	-	-	-	-	-
Divergent_MKVAVVLLVS	1 (1)	3 (4)	-	-	-	-	-	-	1 (1)	-	-	-
Divergent_MRCLSIFVLL	-	4 (5)	-	-	-	-	-	-	-	-	-	-
Divergent_MRFLHFLIVA	-	2 (2)	-	-	-	-	-	-	-	-	-	-
Divergent_MRFYIGLMAA	-	2 (5)	-	-	-	-	-	-	-	-	-	-
Divergent_MSKLVILAVL	-	4 (6)	-	-	-	-	-	-	-	-	-	-
Divergent_MTAKATLLVL	-	2 (2)	-	-	-	-	-	-	-	-	-	-
Divergent_MTLTFLLVVA	-	2 (2)	-	-	-	-	-	-	-	-	-	-
E	4 (4)	2 (2)	2 (3)	-	1 (1)	2 (2)	4 (4)	2 (3)	1 (1)	2 (2)	2 (2)	1 (1)
F	3 (3)	-	4 (4)	2 (3)	-	-	4 (4)	-	3 (3)	3 (3)	2 (2)	1 (1)
G-like	2 (2)	-	-	-	1 (1)	-	-	-	-	2 (2)	-	-
H	-	-	-	1 (1)	-	2 (2)	3 (3)	-	1 (1)	-	-	-
I1	15 (21)	10 (10)	2 (2)	-	2 (2)	-	2 (2)	-	-	11 (11)	2 (2)	-
I2	1 (3)	6 (6)	9 (9)	1 (1)	2 (2)	3 (4)	-	5 (5)	2 (2)	2 (2)	3 (3)	10 (14)
I3	8 (9)	-	2 (2)	2 (2)	-	-	-	-	-	3 (4)	14 (16)	-
I4	4 (4)	-	8 (10)	-	-	1 (1)	3 (4)	-	-	3 (3)	-	-
J	9 (9)	-	10 (13)	-	-	3 (3)	-	1 (1)	-	3 (5)	1 (1)	-

Superfamily	*arenatus*	*californicus*	*coronatus*	*ebraeus*	*imperialis*	*lividus*	*marmoreus*	*quercinus*	*rattus*	*sponsalis*	*varius*	*virgo*
K	-	-	-	-	5 (5)	-	-	-	-	-	2 (2)	-
L	7 (8)	7 (7)	6 (6)	2 (2)	-	11 (14)	-	2 (2)	6 (6)	1 (1)	2 (3)	-
M	8 (10)	6 (6)	41 (53)	22 (23)	6 (8)	21 (26)	18 (20)	8 (9)	5 (5)	29 (30)	18 (23)	4 (7)
MEFRR[r]	2 (2)	-	-	2 (2)	-	5 (5)	-	1 (2)	4 (4)	3 (3)	-	3 (3)
MEVKM[r]	-	1 (1)	-	-	-	-	-	-	-	-	-	-
MKFLL[r]	-	13 (18)	-	1 (1)	-	1 (1)	-	2 (2)	-	2 (2)	1 (1)	1 (1)
MKISL*	1 (1)	-	-	-	1 (1)	1 (2)	-	1 (1)	-	1 (1)	-	1 (1)
MKIVL[r]	-	2 (2)	-	-	-	-	-	-	-	-	-	-
MMLFM[r]	1 (1)	-	13 (13)	-	4 (4)	2 (2)	-	-	2 (2)	-	6 (8)	-
MNCYL[r]	-	7 (8)	-	-	-	-	-	-	-	-	-	-
MRFYM*	2 (2)	-	1 (1)	-	-	-	-	-	2 (2)	-	-	-
MTFYL[r]	-	2 (2)	-	-	-	-	-	-	-	-	-	-
MTSTL[r]	-	1 (1)	-	-	-	-	-	-	-	-	-	-
N	4 (4)	14 (15)	8 (9)	5 (5)	1 (1)	4 (4)	2 (2)	1 (1)	2 (5)	8 (8)	8 (11)	4 (6)
O1	52 (70)	33 (36)	56 (66)	4 (4)	8 (9)	22 (25)	13 (13)	12 (12)	8 (11)	95 (123)	16 (22)	19 (26)
O2	26 (37)	1 (1)	18 (21)	1 (1)	5 (5)	9 (12)	4 (5)	6 (8)	3 (3)	28 (31)	5 (5)	8 (13)
O3	7 (10)	2 (2)	3 (3)	1 (1)	-	1 (1)	-	1 (1)	-	6 (6)	2 (2)	1 (1)
P	13 (17)	-	8 (9)	1 (1)	11 (12)	4 (4)	-	-	2 (2)	13 (16)	13 (14)	-
Q	1 (1)	-	2 (2)	2 (2)	-	9 (11)	-	7 (9)	-	1 (1)	-	1 (2)
S	3 (4)	-	-	-	1 (1)	-	1 (1)	-	1 (1)	-	4 (5)	-
SF-04	2 (2)	-	1 (1)	-	-	2 (2)	-	1 (1)	-	1 (2)	-	1 (1)
SF-mi1	2 (2)	-	8 (10)	1 (2)	-	-	-	-	1 (2)	6 (6)	-	1 (1)
SF-mi2	-	-	-	2 (2)	-	1 (1)	-	2 (4)	2 (3)	3 (3)	3 (3)	1 (1)
T	12 (17)	6 (8)	18 (21)	-	8 (8)	15 (17)	12 (20)	1 (1)	1 (3)	56 (72)	17 (19)	4 (13)
U	-	-	2 (2)	-	-	2 (2)	-	4 (5)	1 (1)	6 (9)	-	1 (1)
V	3 (4)	-	3 (3)	1 (1)	-	21 (28)	-	4 (6)	-	3 (3)	-	2 (3)
Y	1 (2)	-	-	1 (1)	-	1 (2)	-	1 (1)	-	3 (5)	-	2 (2)

- indicates no conotoxins identified.

* indicates a novel gene superfamily.

[r] indicates reclassification of previously identified conotoxins.
