# Supplementary material for: Dietary breadth is positively correlated with venom complexity in cone snails
Source: BMC Genomics. 2016 May 26;17:401. doi: 10.1186/s12864-016-2755-6 (PMC4880860; doi:10.1186/s12864-016-2755-6)
Supplement: Additional file 3: Table S3. — Comparison between final conotoxin dataset and conotoxins assembled through three iterations of Trinity. Number and percent of unique precursor peptides from the final dataset that had at least 95 % sequence identity to transcripts assembled by trinity and that matched to (a) 90 %, (b) 95 %, or (c) 99 % of the total precursor sequence length. (PDF 61 kb) [file 12864_2016_2755_MOESM3_ESM.pdf]

Table S3. Comparison between final conotoxin dataset and conotoxins assembled through three iterations of Trinity. Number and percent of unique precursor peptides from the final dataset that had at least 95% sequence identity to transcripts assembled by trinity and that matched to (a) 90%, (b) 95%, or (c) 99% of the total precursor sequence length.

Species	No. of unique conotoxin precursors; final dataset	(a) 90% of the length		(b) 95% of the length		(c) 99% of the length	
		Total	Frequency	Total	Frequency	Total	Frequency
arenatus	326	225	69.0%	217	66.6%	206	63.2%
californicus	185	159	85.9%	156	84.3%	147	79.5%
coronatus	331	217	65.6%	206	62.2%	200	60.4%
ebraeus	75	65	86.7%	65	86.7%	65	86.7%
imperialis	70	51	72.9%	49	70.0%	48	68.6%
lividus	244	171	70.1%	161	66.0%	156	63.9%
marmoreus	81	70	86.4%	67	82.7%	64	79.0%
quercinus	97	90	92.8%	90	92.8%	90	92.8%
rattus	102	93	91.2%	91	89.2%	89	87.3%
sponsalis	401	291	72.6%	262	65.3%	227	56.6%
varius	198	146	73.7%	138	69.7%	130	65.7%
virgo	113	99	87.6%	99	87.6%	93	82.3%
