# Supplementary material for: Dietary breadth is positively correlated with venom complexity in cone snails
Source: BMC Genomics. 2016 May 26;17:401. doi: 10.1186/s12864-016-2755-6 (PMC4880860; doi:10.1186/s12864-016-2755-6)
Supplement: Additional file 4: Table S4. — Conopeptide gene superfamilies that were reclassified and provided new names in this study. (PDF 66 kb) [file 12864_2016_2755_MOESM4_ESM.pdf]

Table S4. Conopeptide gene superfamilies that were reclassified and provided new names in this study.

New gene superfamily classification	Previous gene superfamily classification/unclassified protein
B4	conantokin-L-like conotoxin, *Conus eburneus* (Genbank #ADZ99323.1)
MEFRR	Divergent_M---L-LTVA
MEVKM	Kappa-conotoxin-like as14a, *Conus austini* (Genbank #P0C6S2)
MKFLL	Divergent_M---L-LTVA
MTFYL	Divergent_M---L-LTVA
MKIVL	Divergent_M---L-LTVA
MMLFM	Putative conotoxin, *Conus caracteristicus* (Genbank #B0L0Y6)
MNCYL	Divergent_M---L-LTVA
MTSTL	Conotoxin Cl9a, *Conus californicus* (Genbank #P0DJB6)
