# Supplementary material for: Dietary breadth is positively correlated with venom complexity in cone snails
Source: BMC Genomics. 2016 May 26;17:401. doi: 10.1186/s12864-016-2755-6 (PMC4880860; doi:10.1186/s12864-016-2755-6)
Supplement: Additional file 5: Figure S1. — New conotoxin gene superfamilies described in this study. Signal sequences are underlined, mature toxin regions are bolded, and cysteines within the mature toxin region are highlighted. Ar = C. arenatus, Co = C. coronatus, Im = C. imperialis, Li = C. lividus, Qc = C. quercinus, Rt = C. rattus, Sp = C. sponsalis, Vi = C. virgo. (PDF 31 kb) [file 12864_2016_2755_MOESM5_ESM.pdf]

MKISL superfamily

```
Ar_MKISL_1    -MKTGLIICLLLIALMIDGGSPGNTMYSRKGAGIASGIKRFQKSFLRRSCTDCPEEPCCYGDQCVADPGHEPFCGN
Im_MKISL_1    -MKTGMIICLLLIAFMDADGSPGDTLYSQKTADTDSGMKRFQKTFQKRRCVFCPKEPCCDGDQCMTAPGTGPFCG-
Li_MKISL_1    -MKISLIICLLLIAFMNGDGSPGNTMYSRKAKDVAAAIKRFQKNFLKKSCTDCPEEPCCFGDQCMPDPGYEPFCGN
Li_MKISL_2    -MKISLIICLLLVAFMNGDGSPGNTMYSRKAKEVAAAIKRFQKNFLKKSCTDCPEEPCCFGDQCMPDPGYEPFCGN
Qc_MKISL_1    -MKISLIICLLLIAFMNGDGSPGNTMYSRRSAGVAAAIKRFQKNFLKKSCNNCPEEPCCAGDQCMPDPGYEPFCGN
Sp_MKISL_1    -MKIGLIICLLLIAFMKGDGSPGKTMYSPKGTGTAFGIKRFQKTFLRRSCNNCPKEPCCYGDQCRSDPGHEPYCGN
Vi_MKISL_1    MMKIGLIICLLLIAFMNGDGSPGNTMRSRKLAGVAAAIKRFQKNFLRRSCNNCPEEPCCAGDQCMADPGYEPFCGN
```

MRFYM superfamily

```
Ar_MRFYM_1     MRFYMLLAVALLLNSVMSTDDVSIRQTD------------ATRRTNPDNPCPPSLPHYCL--SLKADTCCRSECDSGNRCNSNTYKIWR-
Ar_MRFYM_2     MRFYMLLAVALLLNSVMSTDDVSVRQTD------------ATRRTDPDNPCPPSLPHYCL--SLKADTCCRSECDSENRCNSNTYKIWR-
Co_MRFYM_1     MRFYMLLAVALLLNSVMSTDDVSVDEK-------------RKKRDDDNPCPENVPHYCL--NLKADTCCKTACDSSNRCTSKTYEIWR-
Rt_MRFYM_1     MRVYMLLAVALLLNSEMSTDGASFRRQTRARARLGSIADLVRQRKEQDARCPPNYPSVCVYKHRRSYVCCQVPCTV-NSCPIRYSLTIVT
Rt_MRFYM_2     MRVYMLLAVALLLNSEMSTDGASFRRQTRARARLGSIADLVRQRKEQDARCPPTYTSVCVYKHRRSYVCCQVPCTV-NSCPIRYSLTIVT
```
