# Supplementary material for: Dietary breadth is positively correlated with venom complexity in cone snails
Source: BMC Genomics. 2016 May 26;17:401. doi: 10.1186/s12864-016-2755-6 (PMC4880860; doi:10.1186/s12864-016-2755-6)
Supplement: Additional file 6: Table S5. — Cysteine frameworks identified in this study for each gene superfamily. * indicates novel cysteine framework discovered in this study. a indicates a cysteine framework found in cone snail venoms, but not yet described from this gene superfamily. (PDF 71 kb) [file 12864_2016_2755_MOESM6_ESM.pdf]

Table S5. Cysteine frameworks identified in this study for each gene superfamily. * indicates novel cysteine framework discovered in this study. [a] indicates a cysteine framework found in cone snail venoms, but not yet described from this gene superfamily.

Superfamily	Cysteine framework
A	C-C[a], C[a], I, III, VI/VII, noCys[a]
B1	C-C, noCys
B2	noCys
B4	C, noCys
C	C-C
con-ikot-ikot	C-C-C-C-C-CC-C-C-C-C-C*,
	C-CC-C-C-C-C-C-C-CC-C-C-C,
	C-CC-C-C-C-C-C-CC-C-C-C*,
	CC-C-C-C-C-C-C-C-C-C-C-C-C-C-C*,
	CC-C-C-C-C-C-C-C-C-C-C-C-CC-C-C-C*,
	CC-C-C-C-C-C-C-C-C-C-C-C-CC-C-C-C-C-C,
	CC-C-C-C-C-C-C-CC-C-C-C-C-C*,
	CC-C-C-C-C-C-C-CC-C-C-C-C-CC-C-C-C*,
	CC-C-C-C-C-C-C-CC-C-C-C-C-C,
	CC-C-C-C-C-C-CC-C-C-C-C,
	CC-C-C-C-C-C-CC-C-C-C-C-C*,
	CC-C-C-C-C-CC-C-C-C-C-C*,
	CC-C-C-C-CC-C-C-C-C-C-C*, V[a], XXI
conkunitzin	C-C-C-C-C-C-C-C-C-C-C-C,
	C-C-C-C-C-C-CC-C-C-C-C-C-C,
	C-C-C-C-C-C-C,
	C-C-C-C-C-CC-C,
	C-C-C-C-CC-C-C-C-C-C-C-CC-C*, IX, XIIa, XIV
conodipine	C-C-C-C-C-C-C-C-C-C-C-C,
	C-C-C-C-C-C-C-C-C-C-C[a]
conohyal	C-C-C-C-C-C-C-C-C-C-C-C-C*
conophysin	C-C-C-C-C-CC-C-C-C-C-C-CC-C-C*,
	C-C-C-CC-C-C-C-C-C-CC-C-C
conoporin	C, C-C-C[a], C-C[a]
D	C-C-C-CC-C-C-C-C-C,
	C-CC-C-CC-C-C-CC*,
	CC-C-CC-C-C-C-C[a], XX
DivMKFPLLFISL	C-C-C-CC-C-Ca, VI/VII
DivMKLCVVIVLL	XIV
DivMKLLLTLLLG	VIII
DivMKVAVVLLVS	XIV
DivMRCLSIFVLL	XVI, noCys[a]
DivMRFLHFLIVA	C-C[a], VI/VII
DivMRFYIGLMAA	V
DivMSKLVILAVL	IX
DivMTAKATLLVL	XIV
DivMTLTFLLVVA	VI/VII
E	C-C-C-C-C-C-C-C-C[a], IX[a], XXII

Superfamily	Cysteine framework
F	C-C, C[a]
G-like	XX
H	VI/VII
I1	C-C-CC-CC-C-C-C-C[a], C-C-C[a], VI/VII, XI
I2	C-C-CC-CC-C[a],
	C-C-CC-CCC-C-C*, C-CC-CC-C-C[a], VI/VII[a], XI
I3	C[a], VI/VII, XI, noCys[a]
I4	C-C-C-C-CC-C-C-C-C,
	C-C-C-C-CCC-C-C-C*,
	C-C-C-CC-C-C, VI/VII, XII
J	C-C[a], C[a], XIV, noCys[a]
K	C-C[a], XXIII
L	XIV, noCys[a]
M	C-C,
	C-C-C-CC,
	C-C-C-CC-C-C-C-C-C[a],
	C-CC-C-C-C,
	CC-C-C-C,
	CC-C-C-CC-C,
	CC-C-CC,
	I, II, III, IV, IX, XIII[a], XIV, XVI, noCys
MEFRR	C-C-C-CCC-C-C, IX
MEVKM	XIV
MKFLL	C-C-CC-C-C-C-C-CC-C-C*,
	VI/VII, XIV, noCys
MKISL	VI/VII
MKIVL	XIV
MMLFM	C-C-C-C-C-C-C, C-C-C-C-C, VIII
MNCYL	IX, XIII
MRFYM	VI/VII
MTFYL	VI/VII
MTSTL	IX
N	C-C-CC-CC-C-C-C-C,
	IX, XIX, XV, XXII, noCys
O1	C-C-C-C-C,
	C-C-C-C-C-CC-C-C[a],
	C-C-C-CC-C-C,
	C-C-CC-C-C-C,
	C-C-CC-C-CC,
	C-C-CC-CC-C-C-C-C[a],
	C-C-CC-CC-C[a],
	C-C[a],
	CCC-C-CC-C-C,
	I, VI/VII, XI, XII, noCys
O2	C-C,
	C-C-C-CC-C-C,

Superfamily	Cysteine framework
O2	C-C-C[a],
	VI/VII, XIV, XV, noCys[a]
O3	VI/VII, XIV[a]
P	C-C-C-C-C-C-C[a], C-C[a], IX, noCys[a]
Q	C-C-C-CC-C-C[a], C-C[a], III[a], VI/VII, XVI
S	VIII
SF-04	XIII
SF-mi1	XIII
SF-mi2	C-C-C-CCC-C-C
T	C, C-C, CC[a], I, V, XIV[a], XVI[a], noCys
U	C-C[a], VI/VII, XII[a]
V	CC-C-CC-C-C-C-C[a], VI/VII, XV
Y	XVII
