# Supplementary material for: Dietary breadth is positively correlated with venom complexity in cone snails
Source: BMC Genomics. 2016 May 26;17:401. doi: 10.1186/s12864-016-2755-6 (PMC4880860; doi:10.1186/s12864-016-2755-6)
Supplement: Additional file 7: Table S6. — Conopeptides sequenced in this study with 100 % protein sequence identity to a different species on ConoServer. Matches ≥95 % are shown for conopeptides described Conus flavidus to highlight similarities with the C. lividus transcriptome from this study. (PDF 76 kb) [file 12864_2016_2755_MOESM7_ESM.pdf]

Table S6. **Conopeptides sequenced in this study with 100% protein sequence identity to a different species on ConoServer.**
Matches ≥95% are shown for conopeptides described *Conus flavidus* to highlight similarities with the *C. lividus* transcriptome from this study.

Species	Conotoxin identified in this study	Species	Previously published conotoxin	Identity	Gene superfamily	Genbank #
Conus arenatus	Ar_I2_2	*Conus pulicarius*	Pu11.10	100	I2	NA
Conus arenatus	Ar_J_9	*Conus planorbis*	PlXIVA	100	J	Q0N4U8
Conus arenatus	Ar_O1_63	*Conus pulicarius*	Pu6.6	100	O1	NA
Conus arenatus	Ar_O2_22	*Conus pulicarius*	contryphan-like	100	O2	NA
Conus arenatus	Ar_O2_24	*Conus pulicarius*	contryphan-like	100	O2	NA
Conus ebraeus	Eb_M_37	*Conus capitaneus*	Cp3-2-R02	100	M	AEX60040
Conus ebraeus	Eb_M_39	*Conus capitaneus*	Cp3-D03	100	M	AEX60028
Conus imperialis	Im_D_2	*Conus litteratus*	Lt15.5	100	D	ADZ76489
Conus imperialis	Im23a	*Conus marmoreus*	Mr23a	100	K	AFE82855
Conus imperialis	Im23b	*Conus quercinus*	Qc23a	100	K	AFE82857
Conus imperialis	Im23b	*Conus virgo*	Vi23a	100	K	AFE82856
Conus imperialis	ImIIA	*Conus bandanus*	Bn1.3	100	A	P0C1Y2
Conus lividus	Li_A_58	*Conus flavidus*	Fla6.1	100	A	JX499073.1
Conus lividus	Li_A_58	*Conus flavidus*	Fla6.2	100	A	JX499135.1
Conus lividus	Li_A_64	*Conus flavidus*	Fla1.1	100	A	JX499074.1
Conus lividus	Li_A_66	*Conus flavidus*	Fla1.2	100	A	JX499075.1
Conus lividus	Li_A_66	*Conus rattus*	Rt1.1	100	A	AGK23183
Conus lividus	Li_A_67	*Conus flavidus*	Fla1.6	100	A	JX499078.1
Conus lividus	Li_A_69	*Conus flavidus*	Fla1.7	98.77	A	JX499080.1
Conus lividus	Li_A_69	*Conus flavidus*	Fla1.7	98.77	A	JX499079.1
Conus lividus	Li_B1_4	*Conus flavidus*	Fla-4	100	B1	JX499105.1
Conus lividus	Li_B1_5	*Conus flavidus*	Fla-6	98	B1	JX499107.1
Conus lividus	Li_B1_6	*Conus flavidus*	Fla-1	98.67	B1	JX499093.1
Conus lividus	Li_B1_6	*Conus flavidus*	Fla-2	98.98	B1	JX499106.1
Conus lividus	Li_B1_7	*Conus flavidus*	Fla-3	100	B1	JX499094.1
Conus lividus	Li_B1_9	*Conus flavidus*	Fla-5	100	B1	JX499109.1
Conus lividus	Li_B1_9	*Conus flavidus*	Fla-5	100	B1	JX499108.1
Conus lividus	Li_B4_10	*Conus flavidus*	Fla-7	100	B4	JX499110.1
Conus lividus	Li_B4_8	*Conus flavidus*	Fla-8	98.91	B4	JX499111.1
Conus lividus	Li_I2_2	*Conus flavidus*	Fla11.1	100	I2	JX499100.1
Conus lividus	Li_J_3	*Conus flavidus*	Fla-10	100	J	JX499132.1
Conus lividus	Li_J_3	*Conus flavidus*	Fla-11	98.75	J	JX499133.1

Species	Conotoxin identified in this study	Species	Previously published conotoxin	Identity	Gene superfamily	Genbank #
Conus lividus	Li_J_3	*Conus flavidus*	Fla-12	98.75	J	JX499134.1
Conus lividus	Li_J_3	*Conus flavidus*	Fla-9	98.75	J	JX499131.1
Conus lividus	Li_L_12	*Conus flavidus*	Fla14.1	100	L	JX499085.1
Conus lividus	Li_L_12	*Conus flavidus*	Fla14.2	98.8	L	JX499086.1
Conus lividus	Li_L_13	*Conus flavidus*	Fla14.3	100	L	JX499087.1
Conus lividus	Li_M_24	*Conus flavidus*	Fla3.1	100	M	JX499096.1
Conus lividus	Li_M_36	*Conus flavidus*	Fla3.4	100	M	JX499103.1
Conus lividus	Li_M_41	*Conus flavidus*	Fla-13	95.59	M	JX499101.1
Conus lividus	Li_O1_25	*Conus flavidus*	Fla6.4	100	O1	JX499084.1
Conus lividus	Li_O1_30	*Conus flavidus*	Fla-16	98.48	O1	JX499089.1
Conus lividus	Li_O1_30	*Conus flavidus*	Fla-17	100	O1	JX499090.1
Conus lividus	Li_O1_43	*Conus flavidus*	Fla6.6	100	O1	JX499092.1
Conus lividus	Li_O2_14	*Conus flavidus*	Fla6.8	100	O2	JX499072.1
Conus lividus	Li_P_3	*Conus flavidus*	Fla9.1	97.78	P	JX499082.1
Conus lividus	Li_Q_3	*Conus flavidus*	Fla6.14	98.84	Q	KC582373.1
Conus lividus	Li_Q_3	*Conus flavidus*	Fla6.15	100	Q	KC582374.1
Conus lividus	Li_Q_3	*Conus flavidus*	Fla6.16	98.84	Q	KC582375.1
Conus lividus	Li_Q_3	*Conus flavidus*	Fla6.17	98.84	Q	KC582376.1
Conus lividus	Li_Q_4	*Conus flavidus*	Fla16.3	98.51	Q	KC582361.1
Conus lividus	Li_Q_4	*Conus flavidus*	Fla16.4	100	Q	KC582362.1
Conus lividus	Li_Q_5	*Conus flavidus*	Fla16.1	98.68	Q	KC582363.1
Conus lividus	Li_Q_5	*Conus flavidus*	Fla16.2	100	Q	KC582364.1
Conus lividus	Li_Q_7	*Conus flavidus*	Fla16.5	98.48	Q	KC582357.1
Conus lividus	Li_Q_7	*Conus flavidus*	Fla16.6	100	Q	KC582358.1
Conus lividus	Li_Q_7	*Conus flavidus*	Fla16.7	96.97	Q	KC582359.1
Conus lividus	Li_Q_7	*Conus flavidus*	Fla16.8	98.46	Q	KC582360.1
Conus lividus	Li_T_10	*Conus flavidus*	Fla5.2	100	T	JX499113.1
Conus lividus	Li_T_9	*Conus flavidus*	Fla5.1	100	T	JX499112.1
Conus lividus	Li_V_1	*Conus flavidus*	Fla6.13	100	V	JX499115.1
Conus lividus	Li_V_14	*Conus flavidus*	Fla6.10	98.75	V	JX499125.1
Conus lividus	Li_V_14	*Conus flavidus*	Fla6.11	100	V	JX499124.1
Conus lividus	Li_V_14	*Conus flavidus*	Fla6.9	97.5	V	JX499123.1
Conus lividus	Li_V_16	*Conus flavidus*	Fla6.12	97.5	V	JX499126.1
Conus lividus	Li_V_25	*Conus flavidus*	Fla15.8	97.44	V	JX499127.1

Species	Conotoxin identified in this study	Species	Previously published conotoxin	Identity	Gene superfamily	Genbank #
Conus lividus	Li_V_26	*Conus flavidus*	Fla15.10	97.18	V	JX499129.1
Conus lividus	Li_V_26	*Conus flavidus*	Fla15.11	100	V	JX499130.1
Conus lividus	Li_V_4	*Conus flavidus*	Fla15.3	96.25	V	JX499117.1
Conus lividus	Li_V_4	*Conus flavidus*	Fla15.4	97.5	V	JX499118.1
Conus lividus	Li_V_4	*Conus flavidus*	Fla15.5	98.75	V	JX499119.1
Conus lividus	Li_V_6	*Conus flavidus*	Fla15.1	98.75	V	JX499116.1
Conus lividus	Li_V_6	*Conus flavidus*	Fla15.2	100	V	JX499120.1
Conus lividus	Li_V_6	*Conus flavidus*	Fla15.6	98.75	V	JX499121.1
Conus lividus	Li_V_9	*Conus flavidus*	Fla15.7	100	V	JX499122.1
Conus lividus	LiCr95	*Conus flavidus*	Fla6.5	100	O1	JX499088.1
Conus lividus	Lv1.4	*Conus flavidus*	Fla1.3	98.46	A	JX499076.1
Conus lividus	Lv1.4	*Conus flavidus*	Fla1.4	100	A	JX499077.1
Conus lividus	Lv1.4	*Conus flavidus*	Fla1.5	100	A	JX499099.1
Conus lividus	Lv1.9	*Conus flavidus*	Fla1.8	100	A	JX499081.1
Conus lividus	Lv3-1-DEG01	*Conus flavidus*	Fla-15	100	M	JX499095.1
Conus lividus	Lv3-V07	*Conus flavidus*	Fla3.2	98.65	M	JX499097.1
Conus lividus	Lv3-V07	*Conus flavidus*	Fla3.3	97.3	M	JX499098.1
Conus lividus	Lv3-V07	*Conus miles*	Mi3-V01	100	M	AEX60154
Conus marmoreus	Mr	*Conus geographus*	G6.2	100	O1	NA
Conus marmoreus	Mr1.2	*Conus miles*	Mi1.1	100	A	AAS99935
Conus quercinus	Qc3.1	*Conus emaciatus*	Ec3-YDG04	100	M	AEX60045
Conus rattus	Rt_O2_6	*Conus miles*	Mi036	100	O2	AKB91374
Conus rattus	Rt_O2_6	*Conus vitulinus*	Vt15b	100	O2	AGK23203
Conus virgo	Vi_I2_7	*Conus emaciatus*	Em11.5	100	I2	C7DQB8
Conus virgo	Vi_I2_8	*Conus emaciatus*	Em11.3	100	I2	NA
Conus virgo	Vi_M_6	*Conus marmoreus*	Mr6	100	M	ACV87166
Conus virgo	Vi_O1_28	*Conus vexillum*	Conotoxin-1	100	O1	Q5K0D1
Conus virgo	Vi6.1	*Conus vexillum*	Conotoxin-2	100	O1	CAH64852
Conus virgo	ViTx	*Conus emaciatus*	Em11.10	100	I2	P0C252
