# Supplementary material for: Dietary breadth is positively correlated with venom complexity in cone snails
Source: BMC Genomics. 2016 May 26;17:401. doi: 10.1186/s12864-016-2755-6 (PMC4880860; doi:10.1186/s12864-016-2755-6)
Supplement: Additional file 8: Figure S2. — Identical mature toxin from two different species. Full conopeptide precursor sequences of identical mature toxins expressed in more than one species. Signal sequences are underlined, mature toxin regions are bolded, and cysteines within the mature toxin region are highlighted. Co = C. coronatus, Vi = C. virgo. (PDF 9 kb) [file 12864_2016_2755_MOESM8_ESM.pdf]

```
Co_O2_13        MGKLTILVLVAAVLLSTQAMV---RDQPADRDAVPRDDNPGGTSGKFIDVLRRSGCPWEPWCG
Co_O2_14        MGKLTILVLVAAVLLSTQAMV---RDQPADRDAVPRDDNPGGTSGQFIDVLRRSGCPWEPWCG
Vi_O2_7         MGKLTILVLVAAVLLSTQVMVQGDRDQPADRDAVPRDDKLGRPLGKFTNVLRRSGCPWEPWCG
```
