# Supplementary material for: Dietary breadth is positively correlated with venom complexity in cone snails
Source: BMC Genomics. 2016 May 26;17:401. doi: 10.1186/s12864-016-2755-6 (PMC4880860; doi:10.1186/s12864-016-2755-6)
Supplement: Additional file 9: Table S7. — Gene superfamilies that represent > 50 % of the conopeptides identified per species. (PDF 64 kb) [file 12864_2016_2755_MOESM9_ESM.pdf]

Table S7. Gene superfamilies that represent > 50% of the conopeptides identified per species.

Species	Gene superfamilies*	No. of gene superfamilies	Percentage relative to total number of gene superfamilies expressed
arenatus	O1, O2, con-ikot-ikot, I1, P, T	6	16.7%
californicus	O1, N, MKFLL, I1, conoporin, MNCYL	6	20.0%
coronatus	O1, M, con-ikot-ikot, O2, T	5	15.6%
ebraeus	M, N, conodipine, O1	4	14.8%
imperialis	P, T, O1, M, K	5	25.0%
lividus	O1, M, V, A, T, con-ikot-ikot	6	19.4%
marmoreus	M, O1, T	3	21.4%
quercinus	O1, M, Q, A, O2	5	20.0%
rattus	con-ikot-ikot, conoporin, O1, conkunitzin, L, M	6	21.4%
sponsalis	O1, T, M	3	8.6%
varius	M, T, O1, I3, P, A	6	20.7%
virgo	O1, I2, O2, T	4	16.0%

*listed in decreasing order
