# Supplementary material for: Dietary breadth is positively correlated with venom complexity in cone snails
Source: BMC Genomics. 2016 May 26;17:401. doi: 10.1186/s12864-016-2755-6 (PMC4880860; doi:10.1186/s12864-016-2755-6)
Supplement: Additional file 10: Table S8. — Gene superfamilies and conotoxins representing major components of each species’ venom expression levels. Divergent is abbreviated as Div. (PDF 75 kb) [file 12864_2016_2755_MOESM10_ESM.pdf]

Table S8. Gene superfamilies and conotoxins representing major components of each species' venom expression levels. Divergent is abbreviated as Div.

Species	Gene superfamilies representing > 50% TPM values*	Sequence of the most highly expressed mature toxin	Names and gene superfamilies of mature toxins that encompass > 50% TPM values*
arenatus	T, con-ikot-ikot	AALEDADMKTEKGFLSSIVGNLGTVGNLVGSV**CC**QITNS**CC**PED	Ar_T_9 (T), Ar_T_7 (T), Ar_T_10 (T), Ar_con-ikot-ikot_22 (con-ikot-ikot), Ar_con-ikot-ikot_10 (con-ikot-ikot), Ar_con-ikot-ikot_1 (con-ikot-ikot), Ar_con-ikot-ikot_18 (con-ikot-ikot), Ar_B2_1 (B2), Ar_con-ikot-ikot_21 (con-ikot-ikot), Ar_L_6 (L), Ar_B1_13 (B1)
californicus	DivMRFYIGLMAA, O1, DivMKLCVVIVLL, N	DPAP**CC**QHPIET**CC**	Cl_DivMRFYIGLMAA_6 (DivMRFYIGLMAA), Cal22a (N), Cal14.1b (DivMKLCVVIVLL), Cl_DivMRFYIGLMAA_5 (DivMRFYIGLMAA), Cal9.1a (conkunitzin), Cl_O1_40 (O1), Cl_DivMKLCVVIVLL_4 (DivMKLCVVIVLL), Cl_MKFLL_11 (MKFLL), Cl1.3 (T), Cl_MTSTL_1 (MTSTL)
coronatus	M, O1	M**CC**PRD**C**HGG**C**N**CC**	Co_M_18 (M), Co_M_54 (M), Co_O2_3 (O2), Co_O1_33 (O1), Co_M_27 (M), Co_T_9 (T), Co_O1_25 (O1), Co_M_55 (M), Co_M_34 (M), Co_M_57 (M), Co_J_1 (J)
ebraeus	M, SF-mi2, con-ikot-ikot	T**C**TGN**C**RL**C**GAI**CCC**EPKV**C**RNNQ**C**IDD	Eb_SF-mi2_2 (SF-mi2), Eb_con-ikot-ikot_1 (con-ikot-ikot), Eb_I3_2 (I3), Eb6.1 (O1), Eb_F_1 (F)
imperialis	T, A, I2	LPGLTSSGDSDESLPFLNTI**CC**WSGA**CC**G	Im5.4 (T), Im_A_8 (A), Im_I2_9 (I2), Im_E_1 (E), Im_A_7 (A)
lividus	O1, M, A, V, B2	S**C**GHSGAG**C**YTRP**CC**PGLH**C**SGGQAGGL**C**V	Li_O1_25 (O1), Li_O1_30 (O1), Lv3-V07 (M), Li_B2_1 (B2), Li_B2_2 (B2), Lv1.4 (A), Li_B1_2 (B1), Li_V_11 (V), Li_A_56 (A), Li_E_1 (E), LiC121 (T), Li_L_13 (L), LiCr95 (O1), Li_Q_1 (Q), Li_N_3 (N), Li_O3_2 (O3), Li_A_58 (A), Li_H_1 (H)
marmoreus	O1, M, T	Q**C**EDVWMP**C**TSNWE**CC**SLD**C**EMY**C**TQI	MaI51 (O2), Mr3.8 (M), MaIr34 (O1), MrIA (T), conomarphin-Mr1 (M), CMrX (T)
quercinus	M, Q	R**CC**RYP**C**PDS**C**HGS**CC**Y	Qc_M_13 (M), Qc_Q_11 (Q), Qc_B2_1 (B2), Qc1.4a (A)
rattus	L, T	NE**C**TAT**C**EPG**C**VGE**C**D	Rt_L_3 (L), Rt_T_3 (T)
sponsalis	O1, T	WSFSTLFHAV**CC**AYPH**C**RFMPG**C**	Sp_A_4 (A), Sp_O1_14 (O1), Sp_O1_32 (O1), Sp_T_24 (T), Sp_T_6 (T), Sp_L_1 (L), Sp_A_3 (A), Sp_O1_31 (O1), Sp_U_5 (U), Sp_O1_15 (O1), Sp_O1_56 (O1), Sp_O2_18 (O2), Sp_T_33 (T), Sp_O2_17 (O2), Sp_O2_26 (O2), Sp_O1_29 (O1), Sp_T_54 (T), Sp_O1_84 (O1), Sp_T_13 (T), Sp_T_53 (T), Sp_T_30 (T), Sp_O1_40 (O1), Sp_O1_117 (O1)
varius	M, O1, B2	G**CC**PIGP**C**LQSV**C**SP**CC**P	Vr3-SP02 (M), Vr_O1_16 (O1), Vr_B2_2 (B2), Vr_B2_1 (B2), Vr_con-ikot-ikot_2 (con-ikot-ikot)
virgo	O1, I2, O2, M	QQQ**CC**EPAW**C**DGG**C**YD**CC**	Vi_M_2 (M), Vi1.6 (A), Vi_O2_10 (O2), Vi_O1_26 (O1), ViTx (I2), ViVA (T), Vi6.1 (O1), Vi_A_3 (A), Vi_T_14 (T), ViXVA (V), Vi_O1_10 (O1)

*listed in descending order
