# Supplementary material for: Dietary breadth is positively correlated with venom complexity in cone snails
Source: BMC Genomics. 2016 May 26;17:401. doi: 10.1186/s12864-016-2755-6 (PMC4880860; doi:10.1186/s12864-016-2755-6)
Supplement: Additional file 11: Table S9. — Relative contribution of each gene superfamily to conotoxin expression. Values calculated as % conotoxin TPM (superfamily TPM/total conotoxin TPM). (PDF 71 kb) [file 12864_2016_2755_MOESM11_ESM.pdf]

Table S9. Relative contribution of each gene superfamily to conotoxin expression. Values calculated as % conotoxin TPM (superfamily TPM/total conotoxin TPM).

Superfamily	*arenatus*	*californicus*	*coronatus*	*ebraeus*	*imperialis*	*lividus*	*marmoreus*	*quercinus*	*rattus*	*sponsalis*	*varius*	*virgo*
A	5.7%	-	3.1%	-	18.5%	9.8%	0.0%	8.2%	-	8.6%	0.9%	10.4%
B1	3.5%	-	1.4%	0.3%	-	7.0%	-	-	-	2.1%	0.2%	0.0%
B2	2.5%	-	-	-	0.3%	8.1%	4.5%	7.1%	-	-	15.6%	-
B4	-	-	0.0%	-	-	2.4%	-	0.3%	5.8%	0.0%	3.8%	-
C	0.0%	-	-	0.0%	-	0.4%	-	-	0.0%	-	-	-
con-ikot-ikot	20.6%	-	0.5%	14.7%	-	3.2%	-	0.1%	3.8%	1.3%	13.5%	1.5%
conkunitzin	0.6%	3.6%	0.1%	0.0%	-	0.2%	0.0%	-	1.6%	0.3%	0.0%	0.6%
conodipine	0.0%	0.0%	0.0%	0.0%	-	-	-	0.1%	0.0%	0.3%	0.5%	0.1%
conohyal	-	0.0%	-	-	-	-	-	-	-	-	-	-
conophysin	0.1%	-	0.0%	0.0%	0.2%	-	-	0.0%	0.0%	0.0%	2.6%	3.7%
conoporin	-	0.4%	0.0%	0.1%	0.9%	0.0%	-	-	0.4%	-	1.9%	-
D	0.0%	-	0.0%	0.0%	4.8%	-	-	-	1.0%	0.3%	-	-
DivMKFPLLFISL	-	2.2%	0.9%	0.1%	0.1%	-	-	-	1.4%	0.5%	0.0%	-
DivMKLCVVIVLL	-	10.5%	-	-	-	-	-	-	-	-	-	-
DivMKLLLTLLLG	-	1.2%	-	-	-	-	-	-	-	-	-	-
DivMKVAVVLLVS	0.0%	0.2%	-	-	-	-	-	-	0.0%	-	-	-
DivMRCLSIFVLL	-	0.8%	-	-	-	-	-	-	-	-	-	-
DivMRFLHFLIVA	-	1.3%	-	-	-	-	-	-	-	-	-	-
DivMRFYIGLMAA	-	20.5%	-	-	-	-	-	-	-	-	-	-
DivMSKLVILAVL	-	0.3%	-	-	-	-	-	-	-	-	-	-
DivMTAKATLLVL	-	1.0%	-	-	-	-	-	-	-	-	-	-
DivMTLTFLLVVA	-	0.7%	-	-	-	-	-	-	-	-	-	-
E	0.1%	0.1%	0.1%	-	6.8%	2.3%	2.0%	0.1%	0.3%	0.1%	0.2%	0.2%
F	0.2%	-	0.2%	12.5%	-	-	0.0%	-	0.8%	0.3%	3.1%	0.2%
G-like	0.0%	-	-	-	0.3%	-	-	-	-	0.0%	-	-
H	-	-	-	0.0%	-	2.6%	2.5%	-	0.2%	-	-	-
I1	2.7%	2.2%	0.4%	-	6.5%	-	1.2%	-	-	0.5%	0.2%	-
I2	0.5%	2.9%	0.9%	2.8%	9.1%	0.9%	-	0.0%	3.0%	0.0%	0.1%	12.3%
I3	1.3%	-	0.1%	9.2%	-	-	-	-	-	1.3%	1.8%	-
I4	0.5%	-	0.2%	-	-	0.0%	1.3%	-	-	0.0%	-	-
J	3.5%	-	3.7%	-	-	1.1%	-	0.0%	-	0.0%	0.0%	-
K	-	-	-	-	6.7%	-	-	-	-	-	0.2%	-

Superfamily	*arenatus*	*californicus*	*coronatus*	*ebraeus*	*imperialis*	*lividus*	*marmoreus*	*quercinus*	*rattus*	*sponsalis*	*varius*	*virgo*
L	5.1%	2.9%	0.2%	0.0%	-	3.4%	-	1.7%	36.7%	2.8%	0.0%	-
M	0.7%	3.3%	37.9%	21.9%	4.8%	11.7%	21.3%	32.9%	3.3%	2.9%	26.6%	11.8%
MEFRR	0.0%	-	-	4.8%	-	0.3%	-	0.2%	0.9%	0.1%	-	0.2%
MEVKM	-	0.0%	-	-	-	-	-	-	-	-	-	-
MKFLL	-	5.2%	-	0.0%	-	0.0%	-	0.4%	-	0.0%	0.3%	1.1%
MKISL	0.1%	-	-	-	0.2%	0.0%	-	0.6%	-	0.3%	-	0.1%
MKIVL	-	0.0%	-	-	-	-	-	-	-	-	-	-
MMLFM	0.0%	-	0.3%	-	0.3%	1.9%	-	-	0.9%	-	1.1%	-
MNCYL	-	5.8%	-	-	-	-	-	-	-	-	-	-
MRFYM	0.0%	-	0.0%	-	-	-	-	-	2.3%	-	-	-
MTFYL	-	2.1%	-	-	-	-	-	-	-	-	-	-
MTSTL	-	2.6%	-	-	-	-	-	-	-	-	-	-
N	0.1%	9.8%	0.1%	0.3%	0.0%	2.5%	1.2%	1.7%	0.8%	0.4%	4.5%	3.5%
O1	11.8%	14.7%	20.5%	11.6%	0.7%	17.2%	27.3%	8.3%	8.4%	36.7%	17.7%	21.3%
O2	2.7%	0.0%	14.6%	0.1%	4.2%	0.6%	17.9%	9.7%	3.5%	7.2%	0.7%	11.9%
O3	4.1%	1.1%	2.0%	0.0%	-	1.7%	-	0.1%	-	0.1%	0.1%	0.8%
P	0.4%	-	0.7%	0.0%	5.2%	0.7%	-	-	0.3%	1.6%	1.0%	-
Q	0.1%	-	0.0%	0.5%	-	4.7%	-	20.5%	-	0.7%	-	2.4%
S	1.8%	-	-	-	4.3%	-	0.0%	-	0.1%	-	0.8%	-
SF-04	0.0%	-	0.0%	-	-	0.4%	-	0.6%	-	0.5%	-	0.2%
SF-mi1	0.1%	-	1.1%	0.0%	-	-	-	-	0.9%	0.3%	-	0.9%
SF-mi2	-	-	-	19.7%	-	0.0%	-	4.5%	0.2%	0.1%	0.4%	1.1%
T	30.2%	4.8%	10.7%	-	26.1%	6.1%	20.6%	0.1%	22.9%	25.8%	2.2%	9.6%
U	-	-	0.0%	-	-	1.1%	-	2.0%	0.8%	4.0%	-	1.2%
V	0.9%	-	0.0%	1.1%	-	8.5%	-	0.2%	-	0.7%	-	3.7%
Y	0.0%	-	-	0.1%	-	0.8%	-	0.7%	-	0.0%	-	1.1%

- indicates no conotoxins identified.
