# Supplementary material for: Dietary breadth is positively correlated with venom complexity in cone snails
Source: BMC Genomics. 2016 May 26;17:401. doi: 10.1186/s12864-016-2755-6 (PMC4880860; doi:10.1186/s12864-016-2755-6)
Supplement: Additional file 12: Figure S3. — Pseudogene identified from C. sponsalis. Full precursor peptide sequences from the functional and pseudogenized copy of a mature toxin identified from C. sponsalis (Sp = C. sponsalis). TPM values are shown, signal sequences are underlined, mature toxin regions are bolded, and cysteines within the mature toxin region are highlighted. (PDF 9 kb) [file 12864_2016_2755_MOESM12_ESM.pdf]

Sp_O1_79   MKLTCVLIIAVLFLTAC***LITA**DDSRDKQGYRAVRLRDAMQNSKDSR**TPCREKGQGCTNSALCCPGLRCQGTHTAGKCVEES**   TPM = 1242.59

Sp_O1_87   MKLTCVLIIAVLFLTACQLITADDSRDKQGYRAVRLRDAMQNSKDSR**TPCREKGQGCTNSALCCPGLRCQGTHTAGKCVEES**   TPM = 11.66
