# Supplementary material for: Dietary breadth is positively correlated with venom complexity in cone snails
Source: BMC Genomics. 2016 May 26;17:401. doi: 10.1186/s12864-016-2755-6 (PMC4880860; doi:10.1186/s12864-016-2755-6)
Supplement: Additional file 13: Table S10. — Conotoxin composition overlap values (D). Values calculated by (a) the frequency of conopeptide membership to different gene superfamilies (b) the percent expression level of each gene superfamily. (PDF 62 kb) [file 12864_2016_2755_MOESM13_ESM.pdf]

Table S10. Conotoxin composition overlap values (D). Values calculated by (a) the frequency of conopeptide membership to different gene superfamilies (b) the percent expression level of each gene superfamily.

Species comparison	D_{mature}	$D_{expression}$	diet comparison
arenatus-californicus	0.45	0.25	generalist
arenatus-coronatus	0.70	0.39	vermivore
arenatus-ebraeus	0.34	0.31	vermivore
arenatus-imperialis	0.48	0.42	vermivore
arenatus-lividus	0.57	0.43	vermivore
arenatus-marmoreus	0.46	0.40	molluscivore
arenatus-quercinus	0.51	0.22	vermivore
arenatus-rattus	0.46	0.46	vermivore
arenatus-sponsalis	0.66	0.57	vermivore
arenatus-varius	0.52	0.36	vermivore
arenatus-virgo	0.59	0.36	vermivore
californicus-coronatus	0.46	0.27	generalist
californicus-ebraeus	0.34	0.18	generalist
californicus-imperialis	0.35	0.14	generalist
californicus-lividus	0.32	0.31	generalist
californicus-marmoreus	0.35	0.25	not used in calculations
californicus-quercinus	0.35	0.16	generalist
californicus-rattus	0.36	0.26	generalist
californicus-sponsalis	0.42	0.27	generalist
californicus-varius	0.37	0.26	generalist
californicus-virgo	0.43	0.32	generalist
coronatus-ebraeus	0.46	0.36	vermivore
coronatus-imperialis	0.54	0.26	vermivore
coronatus-lividus	0.62	0.46	vermivore
coronatus-marmoreus	0.57	0.68	molluscivore
coronatus-quercinus	0.54	0.55	vermivore
coronatus-rattus	0.51	0.30	vermivore
coronatus-sponsalis	0.67	0.49	vermivore
coronatus-varius	0.60	0.51	vermivore
coronatus-virgo	0.57	0.61	vermivore
ebraeus-imperialis	0.28	0.09	vermivore
ebraeus-lividus	0.43	0.30	vermivore
ebraeus-marmoreus	0.42	0.33	molluscivore
ebraeus-quercinus	0.40	0.36	vermivore
ebraeus-rattus	0.44	0.21	vermivore
ebraeus-sponsalis	0.40	0.20	vermivore
ebraeus-varius	0.45	0.53	vermivore
ebraeus-virgo	0.43	0.31	vermivore
imperialis-lividus	0.46	0.27	vermivore
imperialis-marmoreus	0.49	0.34	molluscivore
imperialis-quercinus	0.44	0.19	vermivore
imperialis-rattus	0.36	0.36	vermivore
imperialis-sponsalis	0.55	0.45	vermivore

Species comparison	D_{mature}	$D_{expression}$	diet comparison
imperialis-varius	0.62	0.14	vermivore
imperialis-virgo	0.42	0.39	vermivore
lividus-marmoreus	0.41	0.46	molluscivore
lividus-quercinus	0.66	0.47	vermivore
lividus-rattus	0.52	0.32	vermivore
lividus-sponsalis	0.51	0.46	vermivore
lividus-varius	0.59	0.51	vermivore
lividus-virgo	0.48	0.60	vermivore
marmoreus-quercinus	0.39	0.45	molluscivore
marmoreus-rattus	0.30	0.37	molluscivore
marmoreus-sponsalis	0.60	0.59	molluscivore
marmoreus-varius	0.45	0.48	molluscivore
marmoreus-virgo	0.43	0.56	molluscivore
quercinus-rattus	0.40	0.19	vermivore
quercinus-sponsalis	0.51	0.33	vermivore
quercinus-varius	0.52	0.47	vermivore
quercinus-virgo	0.62	0.47	vermivore
rattus-sponsalis	0.41	0.45	vermivore
rattus-varius	0.49	0.26	vermivore
rattus-virgo	0.39	0.33	vermivore
sponsalis-varius	0.54	0.30	vermivore
sponsalis-virgo	0.63	0.56	vermivore
varius-virgo	0.47	0.42	vermivore
