# Supplementary material for: Dietary breadth is positively correlated with venom complexity in cone snails
Source: BMC Genomics. 2016 May 26;17:401. doi: 10.1186/s12864-016-2755-6 (PMC4880860; doi:10.1186/s12864-016-2755-6)
Supplement: Additional file 14: Table S11. — Dietary breadth, measured by Shannon-Wiener’s index (H’), for 10 cone snail species sequenced in this study. (PDF 97 kb) [file 12864_2016_2755_MOESM14_ESM.pdf]

Table S11. Dietary breadth, measured by Shannon-Wiener's index (H′), for several cone snail species sequenced in this study.

Species	Average H′	H′	Location	Citation
arenatus	1.89	1.89	Hawaii	Kohn 1959
californicus	2.82	2.82	California	Kohn 1966
coronatus	1.63	1.15	Lady Elliot Island, Great Barrier Reef, Australia	Marsh 1971
		2.1	Thailand, West Indonesia	Kohn & Nybakken 1975
ebraeus	0.81	0.61	Hawaii	Kohn 1959
		0.29	Maldives	Kohn 1968
		0.89	Chagos Islands	Kohn 1968
		0.31	Lady Elliot Island, Great Barrier Reef, Australia	Marsh 1971
		0.81	Low Isles, Great Barrier Reef, Australia	Marsh 1971
		1.1	Thailand, West Indonesia	Kohn & Nybakken 1975
		1.46	Guam	Chang *et al.* 2015
		1.47	American Samoa	Chang *et al.* 2015
		0.35	Hawaii	Chang *et al.* 2015
imperialis	0.58	0.58	Hawaii	Kohn 1959
lividus	1.65	1.23	Hawaii	Kohn 1959
		2.19	Maldives	Kohn 1968
		1.84	Chagos Islands	Kohn 1968
		2.1	Thailand, West Indonesia	Kohn & Nybakken 1975
		0.9	Enewetak Atoll, Marshall Islands	Kohn 1981
marmoreus	0.97	0.97	Hawaii	Kohn 1959
quercinus	0.39	0.39	Hawaii	Kohn 1959
rattus	0.97	1.2	Hawaii	Kohn 1959
		0.63	Chagos Islands	Kohn 1968
		1.3	Thailand, West Indonesia	Kohn & Nybakken 1975
sponsalis	1.92	1.92	Hawaii	Kohn 1959
