# Supplementary material for: Dietary breadth is positively correlated with venom complexity in cone snails
Source: BMC Genomics. 2016 May 26;17:401. doi: 10.1186/s12864-016-2755-6 (PMC4880860; doi:10.1186/s12864-016-2755-6)
Supplement: Additional file 15: Table S12. — Results from Phylogenetic Generalized Least Squares (PGLS) analysis assessing the relationship between prey breadth (H’) and conotoxin complexity. (PDF 65 kb) [file 12864_2016_2755_MOESM15_ESM.pdf]

Table S12. Results from Phylogenetic Generalized Least Squares (PGLS) analysis assessing the relationship between prey breadth (H') and conotoxin complexity.

Conotoxin complexity	(a) with *C. Californicus* Reg. coeff.	λ	Adjusted R	P value	(b) without *C. californicus* Reg. coeff.	λ	Adjusted R	P value
No. of mature toxins	0.0045	1	0.75	0.00071	0.0049	0	0.82	0.00047
No. of gene superfamilies	0.063	0.861	0.45	0.021	0.061	0	0.49	0.021
No. of cysteine frameworks	0.074	1	0.37	0.036	0.071	0	0.35	0.055
